# COVID-19 response: effectiveness of weekly rapid risk assessments, Italy

**DOI:** 10.2471/BLT.21.286317

**Published:** 2021-11-25

**Authors:** Flavia Riccardo, Giorgio Guzzetta, Alberto Mateo Urdiales, Martina Del Manso, Xanthi D Andrianou, Antonino Bella, Patrizio Pezzotti, Simona Carbone, Tiziana De Vito, Francesco Maraglino, Vittorio Demicheli, Claudio Dario, Enrico Coscioni, Giovanni Rezza, Andrea Urbani, Stefano Merler, Silvio Brusaferro, on behalf of the Italian COVID-19 monitoring group

**Affiliations:** a Istituto Superiore di Sanità, Viale Regina Elena 299, 00161 Rome, Italy.; b Fondazione Bruno Kessler, Trento, Italy.; c Ministry of Health, Rome, Italy.; d Agenzia di Tutela della Salute (ATS) Milano, Milan, Italy.; e Regione Umbria, Perugia, Italy.; f Regione Campania, Naples, Italy.

## Abstract

**Problem:**

After Italy’s first national restriction measures in 2020, a robust approach was needed to monitor the emerging epidemic of coronavirus disease 2019 (COVID-19) at subnational level and provide data to inform the strengthening or easing of epidemic control measures.

**Approach:**

We adapted the European Centre for Disease Prevention and Control rapid risk assessment tool by including quantitative and qualitative indicators from existing national surveillance systems. We defined COVID-19 risk as a combination of the probability of uncontrolled transmission of severe acute respiratory syndrome coronavirus 2 and of an unsustainable impact of COVID-19 cases on hospital services, adjusted in relation to the health system’s resilience. The monitoring system was implemented with no additional cost in May 2020.

**Local setting:**

The infectious diseases surveillance system in Italy uses consistent data collection methods across the country’s decentralized regions and autonomous provinces.

**Relevant changes:**

Weekly risk assessments using this approach were sustainable in monitoring the epidemic at regional level from 4 May 2020 to 24 September 2021. The tool provided reliable assessments of when and where a rapid increase in demand for health-care services would occur if control or mitigation measures were not increased in the following 3 weeks.

**Lessons learnt:**

Although the system worked well, framing the risk assessment tool in a legal decree hampered its flexibility, as indicators could not be changed without changing the law. The relative complexity of the tool, the impossibility of real-time validation and its use for the definition of restrictions posed communication challenges.

## Résumé

**Problème:**

Après avoir pris ses premières mesures de restriction nationales en 2020, l'Italie avait besoin d'une approche solide pour surveiller l'épidémie naissante de maladie à coronavirus 2019 (COVID-19) au niveau régional, et fournir les données permettant de renforcer ou d'alléger les mesures destinées à l'endiguer.

**Approche:**

Nous avons adapté l'outil d'évaluation rapide des risques du Centre européen de prévention et de contrôle des maladies en y intégrant des indicateurs quantitatifs et qualitatifs issus des systèmes de surveillance nationaux existants. Pour définir le risque lié à la COVID-19, nous avons associé la probabilité d'une transmission incontrôlée du coronavirus 2 du syndrome respiratoire aigu sévère, à l'impact immédiat des cas de COVID-19 sur les services hospitaliers, en procédant à des ajustements selon la résilience du système de soins de santé. Le dispositif de surveillance a été mis en œuvre en mai 2020 sans entraîner de coûts supplémentaires.

**Environnement local:**

En Italie, le système de surveillance des maladies infectieuses repose sur des méthodes uniformes de collecte de données dans les provinces autonomes et régions décentralisées à travers le pays.

**Changements significatifs:**

Les évaluations des risques réalisées toutes les semaines avec cette approche ont permis de surveiller l'épidémie à l'échelle régionale du 4 mai 2020 au 24 septembre 2021. L'outil a identifié les dates et lieux susceptibles de connaître une augmentation rapide de la demande en services de soins de santé si aucune mesure supplémentaire de contrôle et de lutte n'était prise dans les trois semaines.

**Leçons tirées:**

Bien que le système ait fonctionné, inscrire l'outil d'évaluation des risques dans un décret législatif a réduit sa flexibilité, car les indicateurs ne pouvaient être modifiés sans réformer la loi. La relative complexité de l'outil, l'impossibilité de procéder à une validation en temps réel et son usage pour imposer des restrictions ont posé des problèmes de communication.

## Resumen

**Situación:**

Tras las primeras medidas nacionales de restricción en Italia en 2020, se necesitaba un enfoque sólido para supervisar la epidemia emergente de la coronavirosis de 2019 (COVID-19) a nivel subnacional y proporcionar datos que informaran sobre el refuerzo o la flexibilización de las medidas de contención de la epidemia.

**Enfoque:**

Se adaptó la herramienta de valoración rápida de riesgos del Centro Europeo para la Prevención y el Control de las Enfermedades, al incluir indicadores cuantitativos y cualitativos de los sistemas nacionales de vigilancia existentes. Se definió el riesgo de la COVID-19 como una combinación de la probabilidad de transmisión descontrolada del coronavirus del síndrome respiratorio agudo grave de tipo 2 y de un efecto no sostenible de los casos de la COVID-19 en los servicios hospitalarios, y se ajustó en relación con la capacidad de recuperación del sistema sanitario. El sistema de supervisión se aplicó sin costes adicionales en mayo de 2020.

**Marco regional:**

El sistema de vigilancia de las enfermedades infecciosas en Italia aplica métodos de recopilación de datos coherentes en todas las regiones y provincias autónomas descentralizadas del país.

**Cambios importantes:**

Las valoraciones semanales de los riesgos mediante este enfoque fueron sostenibles en la supervisión de la epidemia a nivel regional entre el 4 de mayo de 2020 y el 24 de septiembre de 2021. La herramienta proporcionó valoraciones fiables de cuándo y dónde se produciría un rápido aumento de la demanda de servicios sanitarios si no se incrementaban las medidas de contención o mitigación en las tres semanas siguientes.

**Lecciones aprendidas:**

Aunque el sistema funcionó bien, el hecho de enmarcar la herramienta de valoración de los riesgos en un decreto legal dificultó su flexibilidad, ya que los indicadores no se podían modificar sin cambiar la ley. La relativa complejidad de la herramienta, la imposibilidad de validación en tiempo real y su uso para la definición de las restricciones plantearon problemas de comunicación.

## ملخص

المشكلة بعد إجراءات التقييد الوطنية الأولى بإيطاليا في عام 2020، كانت هناك حاجة إلى أسلوب فعّال لرصد الوباء المستجد لمرض فيروس كورونا 2019 (كوفيد 19) على المستوى دون الوطني، وتوفير البيانات لدراسة إمكانية تعزيز أو تخفيف تدابير مكافحة الوباء.

الأسلوب قمنا بتكييف أداة لتقييم المخاطر السريعة تابعة للمركز الأوروبي للوقاية من الأمراض ومكافحتها، وذلك من خلال تضمين مؤشرات كمية ونوعية من أنظمة المراقبة الوطنية الحالية. كذلك قمنا بتعريف مخاطر كوفيد 19 على أنها مزيج من احتمالية الانتقال غير المُقيد لمتلازمة الجهاز التنفسي الحادة الشديدة لفيروس كورونا 2، والتأثير غير المستدام لحالات كوفيد 19 على خدمات المستشفيات، مع تعديلها فيما يتعلق بمرونة النظام الصحي. تم تنفيذ نظام المراقبة دون تكلفة إضافية في مايو/أيار 2020.

المواقع المحلية يستخدم نظام مراقبة الأمراض المعدية في إيطاليا طرقًا متسقة لجمع البيانات عبر المناطق اللامركزية في البلد والمقاطعات المستقلة.

التغيّرات ذات الصلة كانت تقييمات المخاطر الأسبوعية باستخدام هذا الأسلوب مستدامة في رصد الوباء على المستوى الإقليمي، طوال الفترة من 4 مايو/أيار 2020 إلى 24 سبتمبر/أيلول 2021. قدمت الأداة تقييمات موثوقة حول توقيت ومكان حدوث زيادة سريعة في الطلب على خدمات الرعاية الصحية في حالة السيطرة، أو إذا لم يتم زيادة تدابير التخفيف في الأسابيع الثلاثة التالية.

الدروس المستفادة على الرغم من أن النظام قد عمل بشكل جيد، إلا أن صياغة أداة تقييم المخاطر في مرسوم قانوني أعاقت مرونته، حيث لا يمكن تغيير المؤشرات دون تغيير القانون. أدى التعقيد النسبي للأداة، واستحالة التحقق من فعاليتها في الوقت الفعلي، واستخدامها لتعريف القيود، إلى ظهور تحديات في التواصل.

## 摘要

问题 2020 年意大利首次实施全国性限制措施后，需要采取可靠方法以监测新型冠状病毒肺炎（新冠肺炎）疫情在地方层面的蔓延情况，并提供数据以表明是否需要加强或放松疫情控制措施。

方法 通过纳入现有国家监测系统的定量和定性指标，我们调整了欧洲疾病预防和控制中心的快速风险评估工具。我们将新型冠状病毒肺炎风险综合定义为严重急性呼吸系统综合症冠状病毒 2 不受控制传播的可能性以及新型冠状病毒肺炎病例对医院服务的非持续性影响，并根据卫生系统的顺应力进行了调整。2020 年 5 月，在没有产生额外成本的前提下实施了监测系统。

当地状况 意大利传染病监测系统在全国各个分散的地区和自治省统一使用相同的数据收集方法。

相关变化 在 2020 年 5 月 4 日至 2021 年 9 月 24 日期间，使用这种方法开展的每周风险评估在监测区域层面疫情情况方面具有可持续性。该工具能够可靠地评估，如果在接下来的 3 周内没有加强控制或缓解措施，何时何地医疗保健服务需求会迅速增加。

经验教训 尽管该系统运作良好，但将风险评估工具纳入法令范畴限制了其灵活性，因为若不更改法律，则无法变更指标。该工具的相对复杂性、实时验证的不可能性及其在法规限定方面的用途导致产生了沟通挑战。

## Резюме

**Проблема:**

После первых национальных ограничительных мер в Италии в 2020 году потребовался активный подход для мониторинга зарождающейся эпидемии коронавирусной инфекции 2019 года (COVID-19) на субнациональном уровне и для предоставления данных для обоснования усиления или ослабления мер по борьбе с эпидемией.

**Подход:**

Авторы адаптировали инструмент для оперативных оценок рисков Европейского центра по контролю и профилактике заболеваний, включив в него количественные и качественные показатели из существующих национальных систем эпиднадзора. Авторы определили риск COVID-19 как комбинацию вероятности неконтролируемой передачи тяжелого острого респираторного синдрома, вызванного коронавирусом-2, и разрушительного воздействия случаев COVID-19 на больничное обслуживание, которая скорректирована с учетом устойчивости системы здравоохранения. Система мониторинга была внедрена без каких-либо дополнительных затрат в мае 2020 года.

**Местные условия:**

В системе эпиднадзора за инфекционными заболеваниями в Италии используются последовательные методы сбора данных по децентрализованным регионам и автономным провинциям страны.

**Осуществленные перемены:**

Еженедельные оценки рисков с использованием данного подхода регулярно применялись при мониторинге эпидемии на региональном уровне с 4 мая 2020 года по 24 сентября 2021 года. Инструмент обеспечил надежную оценку того, когда и где может произойти быстрое увеличение спроса на медицинские услуги, если меры по борьбе или смягчению последствий не будут усилены в течение следующих 3 недель.

**Выводы:**

Несмотря на то что система работала эффективно, включение инструмента для оценок рисков в юридические постановления ограничивало его гибкость, поскольку показатели не могли быть изменены без изменения закона. Относительная сложность инструмента, невозможность проверки в реальном времени и его использование для определения ограничений создают проблемы коммуникации.

## Introduction

In its first response to the emerging epidemic of coronavirus disease 2019 (COVID-19), the Italian government implemented national restriction measures with strict physical distancing and restrictions on public movements^1^ that were maintained from March to May 2020.^2^ Although followed by a rapid decrease in transmission of severe acute respiratory syndrome coronavirus 2 (SARS-CoV-2),^1^ the control measures had an adverse impact on the economy and society.^3^ To continue and further improve Italy’s preparedness and response capacity,^4^ we used an adapted version of the European Centre for Disease Prevention and Control (ECDC) rapid risk assessment tool^5^^,^^6^ to conduct weekly subnational assessments using data from public health intelligence.

## Local setting

Italy’s health-care system is decentralized at regional level across 21 regions and autonomous provinces that largely differ in terms of size, population density and age structure as well as the financing and delivery of health care.^7^ The infectious diseases surveillance system uses consistent data collection methods across the country, with local health units collecting notifications from clinical services and investigating cases. Local health units transfer the data to regional and autonomous provincial authorities who verify and notify cases to the Ministry of Health and to the Italian national institute of health (*Istituto Superiore di Sanità*; the national centre for research, control and scientific advice on public health).

We designed the Italian COVID-19 risk assessment tool to provide reliable assessments in the different regional contexts. The risk assessments produced for each region and autonomous province were initially used to informally assist regional epidemic responses. Subsequently, in October 2020 the assessments were formally integrated as part of a flexible COVID-19 prevention and control response strategy for the autumn to winter 2020 season.^4^

From the beginning of November 2020,^8^ the Italian government implemented laws to define the need for strengthening or easing of regional interventions to control the epidemic, according to fixed parameters. Until May 2021, the weekly regional risk assessments became one of the parameters used to define the level of restrictions to be implemented. We have shown the effectiveness of this approach in a previous publication.^9^ Higher immunization coverage has decreased the impact of COVID-19 in terms of severe disease and death, and therefore the legal parameters used for mitigation of the pandemic were changed and risk assessments were no longer used. However, these assessments are still produced to date with the same method to monitor the epidemic and are published online.^10^

## Approach

The aim of the risk assessment was to provide a weekly overall categorization of the risk of an uncontrolled and unsustainable SARS-CoV-2 outbreak in each of the 21 Italian regions and autonomous provinces. As per ECDC guidance, we defined risk as a combination of the probability of the health threat and its impact,^5^^,^^6^ described using consolidated risk categories (low, moderate, moderate with high probability of evolving to high, and high). We first assessed the probability and impact separately and then combined them to provide an overall risk, which we then adjusted in relation to an assessment of the health system’s resilience. This resilience element was an innovation not included in the original ECDC framework (see the authors’ data repository).^11^

The three components of the Italian risk assessment tool therefore were: (i) probability (evidence of increased transmission, as a proxy for increasing probability of uncontrolled SARS-CoV-2 transmission in a region or autonomous province); (ii) impact (evidence of unsustainable burden of COVID-19 cases on hospital services); and (iii) resilience (capacity of the public health system to withstand the burden of the pandemic and maintain its functions within the test, track and trace strategy). 

We used three sets of quantitative indicators to assess these components (Table 1).^12^ The first set (data quality output indicators) defined the minimum levels of data completeness to allow the risk assessment; the second set (resilience output indicators) monitored the resilience of public health services in maintaining high levels of testing and contact tracing; the third set (probability and impact result indicators) monitored the probability of an uncontrolled spread of SARS-CoV-2 and of an unsustainable impact of COVID-19 on hospital services. For each indicator, we defined thresholds for alerts.

**Table 1:** Indicators used in the weekly COVID-19 epidemiological monitoring in regions and autonomous provinces, Italy, 2020–2021

Indicator set	Indicator	Threshold	Alert
**Output indicator: data quality **			
Quality of data collected at the national level	1.1. Percentage of symptomatic cases with date of symptom onset reported	≥ 60% of cases with increasing trends^a^	< 60% of cases with increasing trends
	1.2. Percentage of cases admitted to hospital (non-intensive care ward) with a date of admission or transfer reported		
	1.3. Percentage of cases admitted to hospital (intensive care unit) with a date of admission or transfer reported		
	1.4. Percentage of notified cases with the municipality of residence reported		
Ability to test all cases in a timely manner	2.1. Percentage of swabs positive for SARS-CoV-2 infection per month, excluding swabs from screening and re-testing; overall and by setting (local, non-hospital, hospital emergency department, other)	Decreasing percentage of positive swabs in hospital settings or emergency departments. Stable or decreasing positive predictive value of tests	Increasing percentage of positive swabs in hospital settings or emergency departments. Increasing positive predictive value of tests
	2.2. Time between date of symptom onset and date of diagnosis of cases	Weekly median gap ≤ 5 days	Weekly median gap > 5 days
**Output indicator: resilience**			
Adequacy of staff resources for contact tracing, isolation and quarantine	2.4. Number of staff dedicated to contact tracing in each local health unit	(SARS-CoV-2) 1 professional staff member per 10 000 population	< 1 professional staff member per 10 000 population
	2.5. Number of staff dedicated in each unit to the activities for the collection and dispatch of clinical samples to the reference laboratories and monitoring of cases and close contacts placed in quarantine and in isolation, respectively		
	2.6. Number of confirmed cases in the region for which an epidemiological investigation has been carried out, with the search for close and total contacts of new confirmed cases	Increasing number of investigated cases (final target 100%)	Decreasing number of investigated cases (and lower than 90%)
**Result indicator: probability and impact**			
Transmission stability	3.1. Number of cases diagnosed in the last 14 days, reported to the health ministry	Weekly number of diagnosed cases stable or decreasing	Increasing number of diagnosed cases in last 5 days
	3.2. Value of transmissibility parameter based on data from the integrated central surveillance system. Two indicators are used, one based on date of symptom onset and one on date of hospitalization^b^	Regional net reproduction number (Rt) ≤ 1 in all regions and autonomous provinces^c^	Rt > 1 or not assessed
	3.4. Number of cases per date of diagnosis and date of symptom onset reported to the integrated central surveillance system per day	Weekly number of reported cases stable or decreasing	Increasing number of diagnosed cases in last 5 days
	3.5. Number of new SARS-CoV-2 clusters, defined as two or more epidemiologically linked cases or an unexpected increase in the number of cases at a defined time and place	No increase in the number of active clusters in the region (that is, clusters with cases still in isolation or quarantine, or new cases reported during the week)	Increasing number of active clusters in the region (that is, clusters with cases still in isolation or quarantine, or new cases reported during the week)
	3.6. Number of new cases not associated with known chains of transmission	No explicit thresholds (qualitative assessment). If new outbreaks occur, the indicator can monitor the quality of contact tracing. Otherwise, the indicator could represent low transmission in which only sporadic cases are observed (considering undetected circulation in people with few symptoms)	In the presence of outbreaks, the indicator requires an unplanned risk assessment defining whether there is sustained and widespread transmission that requires an escalation of epidemic control measures
Pressure on health-care system	3.8. Bed occupancy rate (percentage of available active hospital beds occupied by COVID-19 patients) in intensive care units for COVID-19 patients	Bed occupancy, intensive care ≤ 30%	Bed occupancy, intensive care > 30%
	3.9. Bed occupancy rate (percentage of available active hospital beds occupied by COVID-19 patients) in non-intensive care wards for COVID-19 patients	Bed occupancy, non-intensive care ≤ 40%	Bed occupancy, non-intensive care > 40%

COVID-19: coronavirus disease 2019; SARS-CoV-2: severe acute respiratory syndrome coronavirus 2.

^a^ A value of at least 50% of cases with increasing trends was considered acceptable between 4 May and 25 May 2020.

^b^ We have previously described the details of the calculations.^1^

^c^ Net reproduction number is the transmissibility potential of the disease at a given time *t* of the disease.^1^

Notes: Cases refer to people with a laboratory confirmed diagnosis of SARS-CoV-2 infection reported to the national surveillance system. Translated with minor adaptations from the Decree of the Italian health ministry, 30 April 2020.^12^ The table only includes the non-optional indicators reported in the original ministerial decree as the other indicators were not relevant either because they were never compiled or because they just had a complementary role to the non-optional indicators here reported.

We assessed probability and impact separately using two dedicated algorithms each composed of three trigger questions requiring a yes/no answer. The first two questions in each algorithm were quantitative while the last question was qualitative.^11^ We compiled the quantitative questions using data from the described indicators (Table 1). To answer the qualitative questions, we activated the national event-based surveillance system^13^ and received weekly declarations from regional public health authorities. More specifically, regional authorities declared respectively if an uncontrolled SARS-CoV-2 transmission that could not be managed locally, or if new clusters of infection in vulnerable settings, were occurring.^11^ Once the assessment of the two algorithms for probability and impact was concluded, a first risk level was defined. We then looked at the resilience indicators. If we detected multiple alerts from the resilience indicators, we automatically scaled up the initial risk to the next risk level.

## Relevant changes

**Fig. 1:**
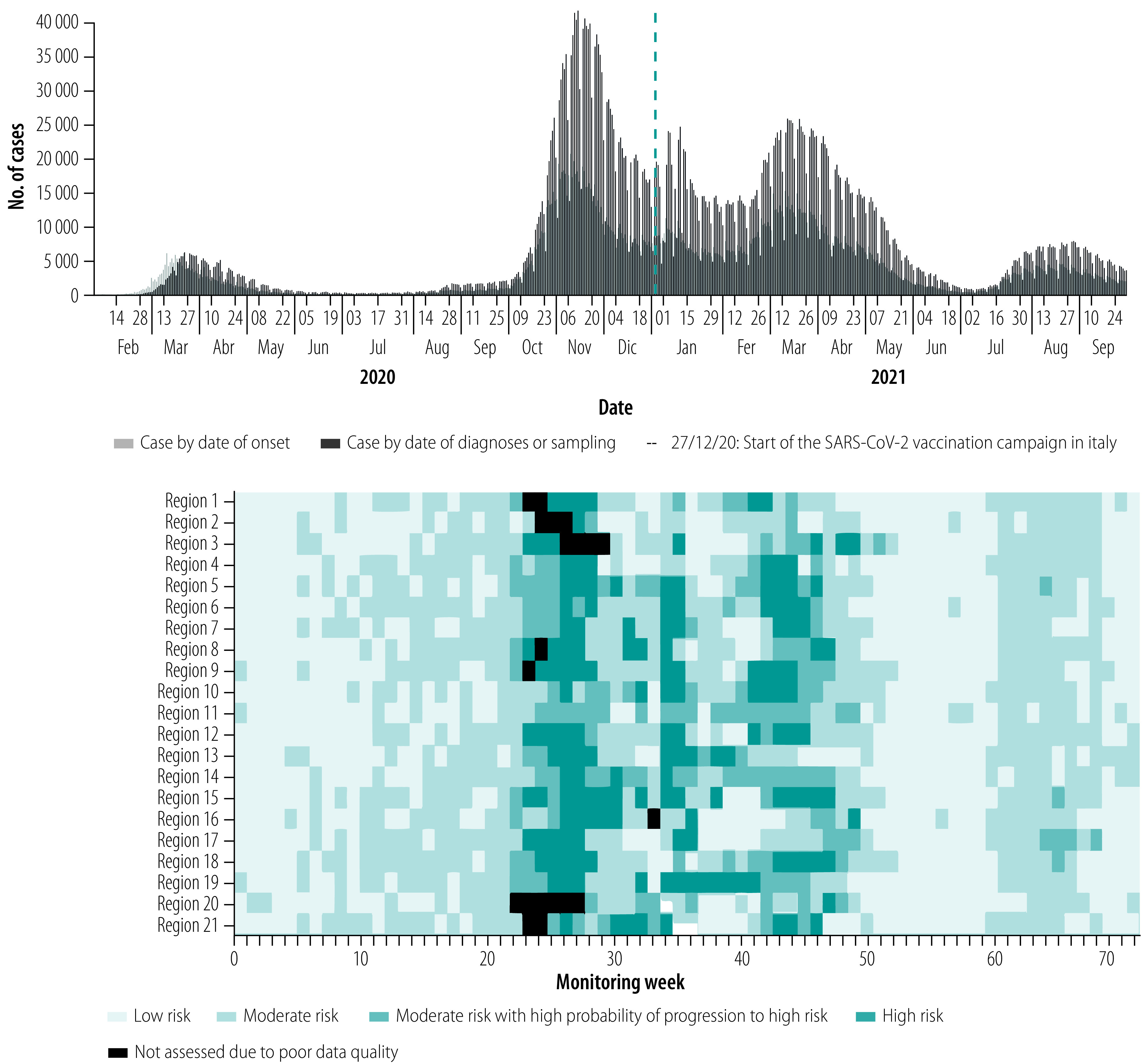
Epidemic curve and weekly risk assessment of the COVID-19 epidemic by region and autonomous province, Italy, 4 May 2020 to 27 September 2021

COVID-19: coronavirus disease 2019; SARS-CoV-2: severe acute respiratory syndrome coronavirus 2.

Note: Cases refer to people with a laboratory confirmed diagnosis of SARS-CoV-2 infection notified to the national integrated case-based SARS-CoV-2 surveillance system. Regions include both regions and autonomous provinces of Italy.

Between 4 May 2020 and 24 September 2021, the Italian national institute of health performed 71 weekly risk assessments.^10^ Each assessment reported an updated classification of risk for each Italian region or autonomous province. As shown in Fig. 1, the risk assessments captured regional risk heterogeneities and were consistent overall with the national epidemic curve. In the data repository,^11^ graphs illustrate how the level of risk assigned was accurate in signalling when increases in the incidence of laboratory-confirmed severe and lethal infections were expected to occur within 3 weeks in the absence of additional control or mitigation measures.

In its early implementation, during low viral circulation in spring to summer 2020, the risk assessment system was very sensitive to localized clusters with limited cases, especially in smaller regions or autonomous provinces. The risk assessment therefore changed occasionally from low to moderate and then back to low as the clusters were contained. These findings, although consistent with the data, were misinterpreted as false alarms and led to some initial concern and distrust in the method among subject-matter experts. As the indicators could not be changed without changing the law, we solved these initial issues by clarifying concerns with public health officials without modifying the risk assessment tool.

Subsequently, the perceived complexity of the tool and the fact that risk assessments always addressed the previous week (too delayed) were criticized.^14^ The net reproduction number (Rt), which reflects the transmissibility of the disease, was also debated and for similar reasons. Even though the risk assessment tool only gave a very limited weight to Rt (Table 1), this controversy targeted its overall validity.

The weekly publication of the risk assessment findings^10^ became a contested topic in the media,^14^ increasingly so between November 2020 and May 2021, when higher risk was automatically associated by law with the enforcement of more severe restrictions to control the spread of the virus. Especially during the autumn to winter 2020 peak of COVID-19 cases, criticism of the assessment system expressed through the media increased, and numerous legal actions were started by representatives of different interest groups and organizations. However, to date, none of the legal actions have led to a re-evaluation of the published risks. Strategies that we adopted to improve public understanding included a weekly presentation of the risk assessments in a press conference and the production of releases and frequently asked questions pages on institutional websites.^15^ The assessment method became less debated after its automatic impact on decision-making stopped.

## Lessons learnt

During a protracted outbreak, ensuring that control measures against the spread of disease are proportionate to the risk is important to limit an unwarranted impact on the economy and on the overall well-being of the population. Ensuring accountability and transparency to the general population is also needed. 

The risk assessment system supported decision-making in Italy by effectively anticipating when the disease outbreak was expected to rapidly worsen, harnessing existing data flows at national and subnational level. The system operated without dedicated funding but, despite requiring a large amount of staff time, was sustainable in the medium term without any disruption in the weekly production of updated risk assessment reports.

Continuous communication between the experts at the national institute of health and public health officials across all the regions and autonomous provinces made sure that the assessments reflected what was happening locally each week while supporting the cohesion of the public health network. Also, different data sources and multiple indicators were helpful in maintaining the robustness of the assessment during increased transmission. 

The main challenge in conducting the risk assessments during the pandemic was related not to the method’s performance but to difficulties in public communication. While we addressed initial misunderstandings among public health officers through technical discussions, the situation changed when the risk assessments started directly impacting restrictions and, consequently, peoples’ daily lives and livelihoods. Criticism of the risk assessment tool (too complex) or of specific parameters (too delayed) stopped being a technical discussion among subject-matter experts and became a contested topic for decision-makers and the general public.

A similar approach to capturing the components of probability, impact and resilience could be adopted in different countries by adapting existing data flows and deploying available human resources. However, we learnt that to increase the acceptability of risk assessment tools like the one described, robustness in performance is not enough (Box 1). Communication issues in applying risk assessment tools should be anticipated during an emergency. Approaches to resolve these issues need to be designed with communication experts, alongside the development of the risk assessment tools, to ensure that the acceptability of the risk assessments is not damaged by controversies driven by misunderstandings.

Box 1 **Summary of main lessons learnt**

- Decision-making during large-scale disease outbreaks can be supported by robust mixed-method risk assessments informed by public health intelligence and existing surveillance systems without dedicated funding.
- Continuous communication with health officials across administrative levels ensures that assessments reflect what is happening in the field and supports the cohesion of the public health network during an emergency.
- Communication issues in applying risk assessment tools should be anticipated during an emergency and approached using dedicated communication tools.
